# Public Awareness and Knowledge of Glaucoma and Cataract: A Cross-Sectional Study

**DOI:** 10.7759/cureus.81928

**Published:** 2025-04-08

**Authors:** Nayef Alswaina, Fatimah M Alayed

**Affiliations:** 1 Department of Ophthalmology, College of Medicine, Qassim University, Buraydah, SAU; 2 College of Medicine, Qassim University, Buraydah, SAU

**Keywords:** cataracts, cross-sectional study, eye health, glaucoma, knowledge, public awareness, saudi arabia

## Abstract

Introduction

Glaucoma and cataracts are the leading causes of visual impairment worldwide. Public awareness and knowledge of these conditions are crucial for early detection and prevention. This study aimed to assess the knowledge level of the general public in the Qassim region of Saudi Arabia regarding glaucoma and cataracts and to evaluate their ability to differentiate between the two conditions.

Method

A cross-sectional study was conducted using a validated online electronic questionnaire distributed via social media platforms. The survey collected demographic data, ocular and medical history, and responses assessing knowledge of glaucoma and cataracts. Statistical analysis was performed using IBM SPSS Statistics for Windows, Version 26.0 (Released 2019; IBM Corp., Armonk, New York, United States), including descriptive statistics and chi-squared tests with a significance level of p<0.05.

Result

A total of 401 participants completed the survey, with 58.4% (n=234) females and 94% (n=377) Saudi nationals. The mean age was 33.36±10.35 years. Most participants (76.6%; n=307) held a bachelor's degree, and 25.2% (n=101) worked in the healthcare field. Only 23.7% (n=95) correctly identified glaucoma as optic nerve damage due to increased eye pressure, while 44.1% (n=177) correctly recognized cataracts as clouding of the lens. The most commonly identified risk factor for glaucoma and cataracts was diabetes (56.4% vs. 53.4%, respectively). Only 23.2% (n=93) correctly associated tunnel vision with glaucoma, while blurry vision, a cataract symptom, was correctly identified by 32.9% (n=132). Higher educational level was significantly associated with better knowledge of cataracts (p=0.002) but not glaucoma (p=0.100). Healthcare professionals had significantly higher knowledge of both conditions (p<0.001).

Conclusion

This study highlights knowledge gaps and misconceptions regarding glaucoma and cataracts among the public in the Qassim region of Saudi Arabia. While cataract awareness was relatively higher, understanding of glaucoma symptoms and risk factors was insufficient. Educational interventions are needed to improve public awareness and promote early detection and prevention strategies for these vision-threatening conditions.

## Introduction

Cataracts and glaucoma are among the most prevalent eye disorders, often coexisting and presenting significant challenges in ophthalmic practice. Cataracts, characterized by the opacification of the lens, lead to progressive visual impairment and typically require surgical intervention for correction. Glaucoma, on the other hand, is a progressive optic neuropathy frequently associated with elevated intraocular pressure (IOP), which, if left untreated, can result in irreversible vision loss. Public awareness and knowledge of these conditions play a crucial role in reducing blindness rates and improving visual health outcomes [1]. However, research suggests significant gaps in awareness, with factors such as education level, socioeconomic status, and access to healthcare influencing the level of knowledge about these eye diseases.

In many regions, awareness of cataracts is considerably higher than that of glaucoma. For instance, a study in Poland found that over 75% of respondents were aware of common eye diseases such as cataracts and glaucoma [2]. In contrast, a study in Jordan reported that only 38.8% of adults recognized glaucoma as a prevalent condition [3]. Additionally, findings from specific health education interventions have demonstrated efficacy in enhancing knowledge; for example, a community-based health campaign in Vietnam resulted in a more than twofold increase in awareness about cataracts and other eye conditions [4]. This emphasizes the potential impact of targeted health education programs in raising awareness and understanding of eye health issues.

Awareness disparities are more pronounced in rural areas and developing countries, where limited healthcare access and inadequate health education contribute to knowledge gaps. A study in Ethiopia found that even health workers in rural areas had poor knowledge of glaucoma, emphasizing the need for improved health education and outreach programs [5]. Integrating eye health education into primary healthcare frameworks is therefore essential to enhance awareness, encourage early diagnosis, and improve treatment outcomes.

Several social and demographic factors influence awareness of glaucoma and cataracts. Studies have shown that age, gender, and educational attainment play critical roles. Older individuals tend to have a higher awareness of glaucoma, with those aged 60-79 years being up to three times more likely to be aware of the disease compared to younger age groups [6,7]. Educational background also plays a crucial role, with individuals who have at least a junior high school education demonstrating significantly better awareness [8,9]. Additionally, media exposure has proven effective in raising awareness, as seen in Ealing, UK, where mass media campaigns, including radio and television broadcasts, successfully improved the public understanding of glaucoma [10].

Gender differences in awareness have also been observed. A study conducted in Taif City, Saudi Arabia, reported that women had significantly higher awareness of glaucoma than men, highlighting the need for targeted interventions to address knowledge disparities, particularly among underrepresented male demographics [11]. Despite some level of awareness, many individuals still lack a deep understanding of glaucoma, which is concerning given its silent progression and irreversible vision loss. A study in Nigeria found that although many people had heard of glaucoma, their comprehension of the disease remained inadequate, suggesting that awareness campaigns should focus not only on recognition but also on providing comprehensive education [12]. Even among glaucoma patients, studies indicate that knowledge gaps persist, which may negatively affect treatment adherence and disease management [13].

Given these findings, addressing public misconceptions and knowledge gaps regarding glaucoma and cataracts is crucial. This study aims to evaluate the level of knowledge of the general public in the Qassim region of Saudi Arabia regarding these two eye conditions and to assess their ability to differentiate between them. By identifying factors influencing awareness levels, this study seeks to provide evidence-based recommendations for enhancing public health education and improving eye care accessibility.

## Materials and methods

Study design and setting

This study utilized a cross-sectional survey design to assess the public's knowledge of glaucoma and cataracts and their ability to differentiate between the two conditions. Data collection was conducted over a three-month period through an online electronic questionnaire (see Appendices) distributed via social media platforms, targeting the general public in the Qassim region of Saudi Arabia.

Study population and sampling

A non-probability convenience sampling technique was employed. The survey was designed to include adults aged 18 years and older, with no restrictions on gender, nationality, or educational background. Participants were required to provide complete responses for inclusion in the study.

Survey instrument and data collection

The electronic questionnaire, developed and validated by Alammar et al. [14], collected demographic information, ocular and medical history, and responses related to glaucoma and cataract knowledge. The survey included multiple-choice questions assessing knowledge of disease characteristics, risk factors, symptoms, and treatment options. The questionnaire was reviewed by medical professionals and distributed online via social media platforms.

Statistical analysis

Data were entered and analyzed using IBM SPSS Statistics for Windows, Version 26.0 (Released 2019; IBM Corp., Armonk, New York, United States). Descriptive statistics, including frequencies and percentages, were used to summarize demographic and categorical variables. Chi-squared tests were performed to assess associations between knowledge levels and demographic factors. A p-value of <0.05 was considered statistically significant.

Ethical considerations

This study was approved by the Regional Research Ethics Committee of Al-Qassim Region, Saudi Arabia (approval number: 607/46/8582). Participation was entirely voluntary, and all participants provided informed consent before completing the survey. They were fully informed about the study's purpose, the voluntary nature of participation, and their right to withdraw at any time without any consequences. To ensure confidentiality, no personally identifiable information was collected, and all responses remained anonymous. The data were securely stored, with access restricted to the research team, adhering to ethical guidelines for confidentiality and data protection.

## Results

A total of 401 participants were included in the study. The majority were female (58.4%; n=234), while 41.6% (n=167) were male. Most participants were Saudi nationals (94%; n=377), with only 6% (n=24) being non-Saudis. The mean age of the participants was 33.36±10.35 years. Regarding education, 76.6% (n=307) held a bachelor's degree, 13.7% (n=55) completed secondary education, and 9.2% (n=37) had postgraduate qualifications. Additionally, 25.2% (n=101) of the participants worked in the healthcare field. Among the participants, 58.6% (n=235) had previously visited an eye clinic, while 41.4% (n=166) had never done so (Table 1).

**Table 1:** Demographic characteristics of the study participants (N=401)

Variable	n (%)
Gender	
Male	167 (41.6%)
Female	234 (58.4%)
Nationality	
Saudi	377 (94%)
Non-Saudi	24 (6%)
Educational level	
Illiterate	1 (0.2%)
Intermediate	1 (0.2%)
Secondary	55 (13.7%)
Bachelor's degree	307 (76.6%)
Postgraduate	37 (9.2%)
Work in healthcare	
Yes	101 (25.2%)
No	300 (74.8%)
Eye clinic visit	
Yes	235 (58.6%)
No	166 (41.4%)

**Figure 1:**
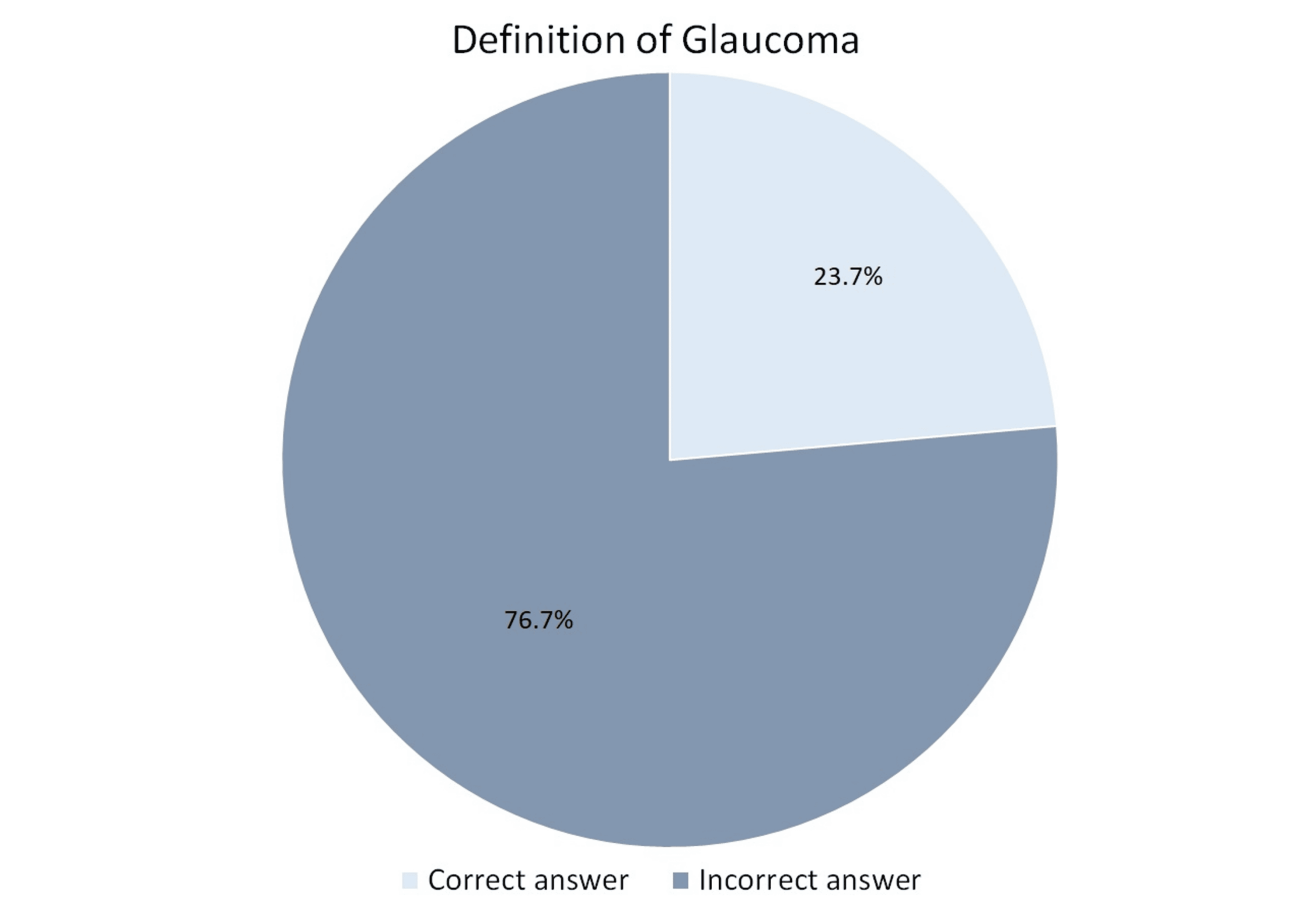
Awareness of the definition of glaucoma among the study participants

A family history of glaucoma or cataracts was reported by 26.9% (n=132) of the participants, and 58.6% (n=235) personally knew someone with either condition. Regarding systemic diseases, 12% (n=48) had diabetes, and 7.2% (n=29) had been diagnosed with high blood pressure. When asked about glaucoma, only 23.7% (n=95) correctly identified it as damage to the optic nerve due to increased eye pressure, whereas 27.4% (n=110) incorrectly believed it to be clouding of the lens, and 27.2% (n=109) stated they did not know the correct answer (Figure 1).

**Figure 2:**
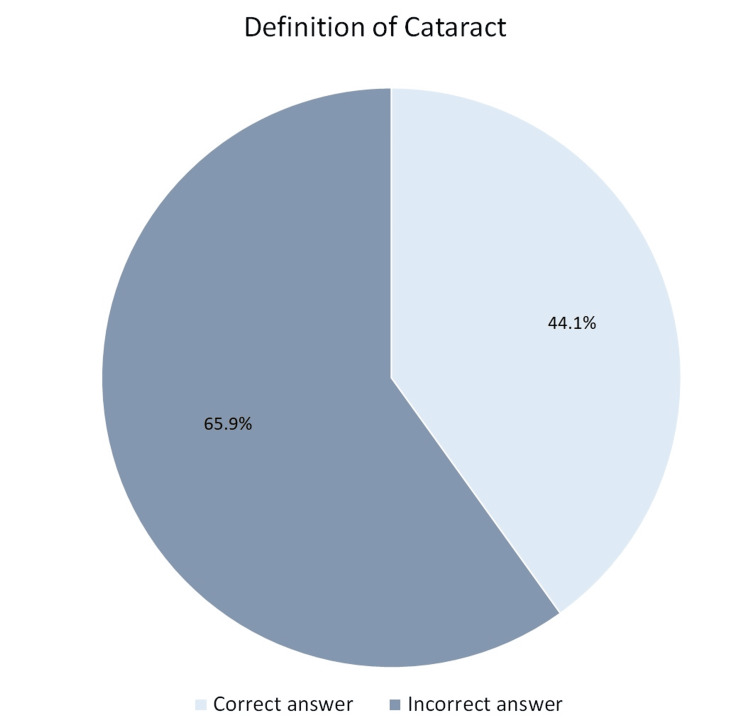
Awareness of the definition of cataract among the study participants

Similarly, for cataracts, 44.1% (n=177) correctly identified it as clouding of the lens, but 16% (n=64) confused it with optic nerve damage, and 20.7% (n=83) were unsure (Figure 2).

The most commonly identified risk factors for glaucoma and cataracts were diabetes (56.4% vs. 53.4%), family history (44.6% vs. 43.1%), and age over 60 years (39.4% vs. 48.4%), respectively (Table 2).

**Table 2:** Participants' awareness of risk factors for glaucoma and cataracts (N=401)

Risk factors	Glaucoma, n (%)	Cataracts, n (%)
Family history	179 (44.6%)	173 (43.1%)
Diabetes	226 (56.4%)	214 (53.4%)
Smoking	63 (15.7%)	63 (15.7%)
Corticosteroid use	74 (18.5%)	59 (14.7%)
Excessive sun exposure	34 (8.5%)	62 (15.5%)
Age over 60 years	158 (39.4%)	194 (48.4%)
Contact lens use	39 (9.7%)	47 (11.7%)
Severe nearsightedness	42 (10.5%)	29 (7.2%)
Dark skin tone	10 (2.5%)	8 (2%)

Knowledge of glaucoma and cataract symptoms varied among participants. When asked about blind spots, only 22.9% (n=92) correctly associated them with glaucoma, while 23.4% (n=94) incorrectly attributed them to cataracts, and 22.7% (n=91) were uncertain. Blurry vision, a common symptom of cataracts, was correctly identified by 32.9% (n=132) of participants, yet 24.4% (n=98) associated it with glaucoma, and 20.4% (n=82) reported not knowing. A high proportion of uncertainty was observed for tunnel vision, a key glaucoma symptom, where 32.9% (n=132) of respondents were unsure, and only 23.2% (n=93) correctly linked it to glaucoma. Similarly, for seeing halos around lights, only 26.2% (n=105) associated it with glaucoma, while 25.9% (n=104) reported not knowing (Table 3).

**Table 3:** Participants' knowledge of glaucoma and cataract symptoms (N=401)

Symptoms	Glaucoma, n (%)	Cataracts, n (%)	I don't know, n (%)
Blind spots	92 (22.9%)	94 (23.4%)	91 (22.7%)
Poor night vision	102 (25.4%)	110 (27.4%)	88 (21.9%)
Blurry vision	98 (24.4%)	132 (32.9%)	82 (20.4%)
Tunnel vision	93 (23.2%)	80 (20%)	132 (32.9%)
Seeing halos around lights	105 (26.2%)	91 (22.7%)	104 (25.9%)

Table 4 shows the awareness of treatment and disease progression among participants. Only 48.1% (n=193) of participants correctly stated that glaucoma can lead to blindness, while 40.1% (n=161) correctly identified that cataracts can cause blindness. Regarding treatment, 40.6% (n=163) correctly stated that glaucoma can be treated with medication, while only 29.9% (n=120) correctly identified that cataracts cannot be treated with medication.

**Table 4:** Participants' awareness of disease progression and treatment options for glaucoma and cataracts (N=401) * indicates true statements

Statement	True, n (%)	False, n (%)	I don’t know, n (%)
Glaucoma can lead to blindness*	193 (48.1%)	86 (21.4%)	122 (30.4%)
Cataracts can lead to blindness*	161 (40.1%)	114 (28.4%)	126 (31.4%)
Vision loss due to glaucoma is reversible	129 (32.2%)	119 (29.7%)	153 (38.2%)
Vision loss due to cataracts is reversible*	194 (48.4%)	79 (19.7%)	128 (31.9%)
Glaucoma can be treated with medication*	163 (40.6%)	76 (19%)	162 (40.4%)
Cataracts can be treated with medication	120 (29.9%)	113 (28.2%)	168 (41.9%)

Chi-squared analysis showed a significant association between educational level and correct identification of cataract (χ²=42.97; p=0.002), with participants holding higher education degrees demonstrating better understanding. However, no statistically significant association was found between educational level and correct identification of glaucoma (χ²=28.42; p=0.100). Participants working in the healthcare field were significantly more likely to correctly identify glaucoma (χ²=120.1; p<0.001) and cataracts (χ²=73.4; p<0.001) compared to non-healthcare workers. Table 5 summarizes the chi-squared analysis of the association between education level and identification of correct symptoms of glaucoma and cataract.

**Table 5:** Association between educational level and knowledge of glaucoma and cataracts * indicates correct answer

Symptoms		Educational level, n					Total (N=401)	P-value (chi-square)
		Illiterate (n=1)	Intermediate (n=1)	Secondary (n=55)	Bachelor's degree (n=307)	Postgraduate (n=37)		
Blind spots	Glaucoma*	1	0	10	73	8	92	0.298
	Cataract	0	0	12	72	10	94	
	Both glaucoma and cataract	0	1	13	65	9	88	
	None of the above	0	0	1	34	1	36	
	I don't know	0	0	19	63	9	91	
Poor night vision	Glaucoma	1	0	13	84	4	102	0.196
	Cataract*	0	1	15	85	9	110	
	Both glaucoma and cataract	0	0	7	52	10	69	
	None of the above	0	0	3	28	1	32	
	I don't know	0	0	17	58	13	88	
Blurry vision	Glaucoma	1	0	10	75	12	98	0.477
	Cataract*	0	1	17	106	8	132	
	Both glaucoma and cataract	0	0	7	46	8	61	
	None of the above	0	0	6	22	0	28	
	I don't know	0	0	15	58	9	82	
Photosensitivity	Glaucoma	1	0	16	88	3	108	0.173
	Cataract	0	1	6	65	6	78	
	Both glaucoma and cataract*	0	0	8	36	9	53	
	None of the above	0	0	6	30	6	42	
	I don't know	0	0	19	88	13	120	
Tunnel vision	Glaucoma*	1	0	13	73	6	93	0.401
	Cataract	0	1	6	66	7	80	
	Both glaucoma and cataract	0	0	9	45	6	60	
	None of the above	0	0	2	29	5	36	
	I don't know	0	0	25	94	13	132	
Shadow or curtain covering the visual field	Glaucoma*	1	0	14	78	7	100	0.787
	Cataract	0	1	11	74	7	93	
	Both glaucoma and cataract	0	0	11	39	6	56	
	None of the above	0	0	4	32	3	39	
	I don't know	0	0	15	84	14	113	
Eye pain	Glaucoma*	1	0	12	86	6	105	0.156
	Cataract	0	0	12	55	8	75	
	Both glaucoma and cataract	0	0	9	55	12	76	
	None of the above	0	1	8	26	4	39	
	I don't know	0	0	14	85	7	106	
Halos around lights	Glaucoma	1	1	13	82	8	105	0.672
	Cataract	0	0	13	73	5	91	
	Both glaucoma and cataract*	0	0	6	56	8	70	
	None of the above	0	0	4	24	3	31	
	I don't know	0	0	19	72	13	104	
Flashes of light	Glaucoma	0	1	13	65	6	85	0.541
	Cataract	0	0	8	67	7	82	
	Both glaucoma and cataract	1	0	7	41	7	56	
	None of the above*	0	0	6	39	7	52	
	I don't know	0	0	21	95	10	126	
Temporary loss of vision	Glaucoma	0	0	10	71	6	87	0.28
	Cataract	0	0	9	55	9	73	
	Both glaucoma and cataract	1	0	10	51	7	69	
	None of the above*	0	1	3	38	4	46	
	I don't know	0	0	23	92	11	126	
Frequent changes in eyeglass prescription measurements	Glaucoma	1	0	10	50	8	69	0.279
	Cataract*	0	0	14	65	9	88	
	Both glaucoma and cataract	0	0	4	55	5	64	
	None of the above	0	0	4	52	7	63	
	I don't know	0	1	23	85	8	117	
Red eye	Glaucoma*	1	0	8	79	7	95	0.452
	Cataract	0	1	11	61	11	84	
	Both glaucoma and cataract	0	0	7	45	7	59	
	None of the above	0	0	8	30	5	43	
	I don't know	0	0	21	92	7	120	
Excessive tearing	Glaucoma	1	1	10	76	4	92	0.153
	Cataract	0	0	9	54	5	68	
	Both glaucoma and cataract	0	0	10	50	6	66	
	None of the above*	0	0	4	29	10	43	
	I don't know	0	0	22	98	12	132	

## Discussion

This study assessed public awareness and knowledge of glaucoma and cataracts in the Qassim region of Saudi Arabia, revealing significant gaps in knowledge, particularly regarding glaucoma and its risk factors and symptoms. Cataracts were more widely recognized, with 44.1% of participants correctly identifying them as clouding of the lens, while only 23.7% correctly defined glaucoma as optic nerve damage due to increased intraocular pressure. Misconceptions were prevalent, with 27.4% of respondents confusing glaucoma with cataracts. Similar knowledge gaps have been reported in previous studies [15,16]. According to a meta-analysis by Morya et al. [17], 57.63% of Saudi citizens had heard or read about cataracts, while 69.9% were aware of glaucoma. In contrast, a study conducted in Iran reported awareness levels of 46.6% for glaucoma and 82.9% for cataracts among the general population [18]. A study conducted in China found that only 1.7% of participants demonstrated a good understanding of cataracts [19].

Diabetes was the most recognized risk factor for both glaucoma (56.4%) and cataracts (53.4%), followed by family history and age over 60 years old. However, modifiable risk factors such as smoking, corticosteroid use, and excessive sun exposure were poorly recognized, with less than 20% of participants correctly identifying them. Similar findings have been previously reported in Saudi Arabia, where public awareness of the role of lifestyle factors in eye disease development remains low [20]. Limited awareness of these modifiable risk factors may contribute to preventable vision impairment, highlighting the need for community-based awareness programs that educate the public on lifestyle-related risks and the importance of routine eye examinations.

Misconceptions were especially prominent in symptom recognition. Only 23.2% of participants correctly associated tunnel vision with glaucoma, while 32.9% were uncertain. Similarly, blurry vision, which is primarily a symptom of cataracts, was correctly identified by 32.9%, yet 20.4% remained unsure. Seeing halos around lights, another key glaucoma symptom, was correctly identified by only 26.2%, while 25.9% reported not knowing. This uncertainty regarding symptoms is concerning, as delayed recognition of warning signs can result in late presentation and irreversible vision loss, a pattern observed in other studies from the region [21]. While it is important to raise awareness of symptoms like peripheral vision loss, educational efforts must also emphasize that glaucoma can progress silently without noticeable symptoms, especially in its early stages. Framing glaucoma as a "silent thief of sight" may better prompt proactive eye check-ups, rather than waiting for visible signs to appear.

Higher educational levels were significantly associated with better knowledge of cataracts (p=0.002) but not glaucoma (p=0.100). This suggests that formal education may contribute to cataract awareness, possibly due to greater exposure to cataract surgery as a common procedure. This is in contrast with the findings of previous studies that found a significant association between higher educational levels and knowledge of glaucoma [7,11]. Healthcare professionals demonstrated significantly higher knowledge of both conditions (p<0.001), reinforcing the role of professional exposure in disease awareness. This finding underscores the importance of extending public health initiatives to non-healthcare individuals, who may be less familiar with preventive measures and early warning signs.

Participants also exhibited significant misconceptions about disease treatment and progression. While 48.1% correctly stated that glaucoma can lead to blindness, 30.4% were unsure. Similarly, only 40.1% correctly identified that cataracts can cause blindness, indicating an underestimation of their potential severity. Regarding treatment, 40.6% correctly identified that glaucoma can be managed with medication, but a substantial 40.4% were uncertain. More concerningly, only 29.9% correctly stated that cataracts cannot be treated with medication, while 41.9% were unsure. This widespread misconception about treatment options suggests that more efforts are needed to educate the public on appropriate management approaches for each condition. Previous studies have similarly documented confusion regarding treatment modalities, emphasizing the importance of health literacy programs that clarify the differences between medical management for glaucoma and surgical correction for cataracts [22].

The findings highlight critical gaps in public awareness, particularly regarding glaucoma symptoms, risk factors, and treatment misconceptions. Given that early detection is crucial for preventing vision loss, targeted public health strategies should focus on improving community awareness through structured health education campaigns, increasing access to eye screenings, and leveraging mass media to disseminate accurate information. Prior research suggests that integrating eye health education into primary healthcare settings and utilizing digital platforms for educational outreach can significantly enhance knowledge and encourage proactive healthcare-seeking behaviors [4].

This study has several limitations. First, the use of an online self-reported questionnaire may have introduced selection bias, as individuals with higher education levels or greater interest in eye health may have been more likely to participate, potentially overestimating public awareness. Second, the study relied on self-reported knowledge, which may not accurately reflect actual understanding, as some participants may have guessed responses or had difficulty recalling information. Third, the cross-sectional design limits the ability to establish causality between demographic factors and knowledge levels. Additionally, the study was conducted in the Qassim region of Saudi Arabia, and findings may not be generalizable to other regions with different healthcare access and awareness levels. Another limitation was that 25.2% of participants worked in the healthcare field, which may introduce a bias in knowledge and awareness levels, potentially leading to an overestimation of overall awareness in the study population. Future research should include longitudinal studies to assess awareness and investigate effective intervention strategies to enhance public education on glaucoma and cataracts.

## Conclusions

This study highlights significant gaps in public knowledge and awareness regarding glaucoma and cataracts among the general population in the Qassim region of Saudi Arabia. While awareness of cataracts was relatively higher, many participants demonstrated poor understanding of glaucoma, particularly its risk factors, symptoms, and irreversible nature. Misconceptions about treatment options were evident, with some participants believing glaucoma could be cured and cataracts could be treated with medication. Higher education and working in healthcare were associated with better knowledge, but even among these groups, misconceptions persisted. The study emphasized the need for structured public health initiatives to improve eye health literacy. Recommendations include targeted awareness campaigns, mass media outreach, and integrating eye health education into primary care services. Future efforts should focus on educating high-risk and underserved populations to enhance the early detection, proper management, and prevention of vision loss.

## Appendices

Section 1: Personal information of participants

1. Gender:

(a) Male

(b) Female

2. Age:

3. Nationality:

(a) Saudi

(b) Non-Saudi

4. Educational level:

(a) Illiterate

(b) Primary

(c) Intermediate

(d) Secondary

(e) Bachelor's degree

(f) Postgraduate

5. Do you work in the healthcare field?

(a) Yes

(b) No

6. Have you ever visited an eye clinic?

(a) Yes

(b) No

7. Do you suffer from any eye diseases?

(a) Yes

(b) No

8. Have you been diagnosed with diabetes?

(a) Yes

(b) No

9. Have you been diagnosed with high blood pressure?

(a) Yes

(b) No

10. Is there a family history of glaucoma or cataracts?

(a) Yes

(b) No

11. Do you know someone who has glaucoma or cataracts?

(a) Yes

(b) No

Section 2: Questions about glaucoma and cataracts

1. Which of the following is correct about glaucoma?

(a) Clouding of the lens

(b) Damage to the optic nerve, often due to increased eye pressure (Correct answer)

(c) Corneal inflammation

(d) Retinal detachment

(e) Increased tearing

(f) I don't know

2. Which of the following is correct about cataracts?

(a) Clouding of the lens (Correct answer)

(b) Damage to the optic nerve, often due to increased eye pressure

(c) Corneal inflammation

(d) Retinal detachment

(e) Increased tearing

(f) I don't know

3. Which of the following is a risk factor for glaucoma?

(a) Family history (Correct answer)

(b) Diabetes (Correct answer)

(c) Smoking (Correct answer)

(d) Corticosteroid use (Correct answer)

(e) Excessive sun exposure

(f) Age over 60 (Correct answer)

(g) Contact lens use

(h) Severe nearsightedness (Correct answer)

(i) Dark skin tone

4. Which of the following is a risk factor for cataracts?

(a) Family history (Correct answer)

(b) Diabetes (Correct answer)

(c) Smoking (Correct answer)

(d) Corticosteroid use (Correct answer)

(e) Excessive sun exposure (Correct answer)

(f) Age over 60 (Correct answer)

(g) Contact lens use

(h) Severe nearsightedness

(i) Dark skin tone

5. Blind spots are a symptom of which of the following conditions?

(a) Glaucoma (Correct answer)

(b) Cataracts

(c) Both glaucoma and cataracts

(d) None of the above

(e) I don't know

Section 3: Symptoms of glaucoma and cataracts

6. Night vision impairment is a symptom of which of the following conditions?

(a) Glaucoma

(b) Cataracts (Correct answer)

(c) Both glaucoma and cataracts

(d) None of the above

(e) I don't know

7. Blurred vision is a symptom of which of the following conditions?

(a) Glaucoma

(b) Cataracts (Correct answer)

(c) Both glaucoma and cataracts

(d) None of the above

(e) I don't know

8. Light sensitivity is a symptom of which of the following conditions?

(a) Glaucoma

(b) Cataracts

(c) Both glaucoma and cataracts (Correct answer)

(d) None of the above

(e) I don't know

9. Tunnel vision is a symptom of which of the following conditions?

(a) Glaucoma (Correct answer)

(b) Cataracts

(c) Both glaucoma and cataracts

(d) None of the above

(e) I don't know

10. A shadow or curtain covering the visual field is a symptom of which of the following conditions?

(a) Glaucoma (Correct answer)

(b) Cataracts

(c) Both glaucoma and cataracts

(d) None of the above

(e) I don't know

11. Eye pain is a symptom of which of the following conditions?

(a) Glaucoma (Correct answer)

(b) Cataracts

(c) Both glaucoma and cataracts

(d) None of the above

(e) I don't know

12. Seeing halos around lights is a symptom of which of the following conditions?

(a) Glaucoma

(b) Cataracts

(c) Both glaucoma and cataracts (Correct answer)

(d) None of the above

(e) I don't know

13. Seeing flashes of light is a symptom of which of the following conditions?

(a) Glaucoma

(b) Cataracts

(c) Both glaucoma and cataracts

(d) None of the above (Correct answer)

(e) I don't know

14. Temporary vision loss for a few seconds is a symptom of which of the following conditions?

(a) Glaucoma

(b) Cataracts

(c) Both glaucoma and cataracts

(d) None of the above (Correct answer)

(e) I don't know

15. Frequent changes in eyeglass prescription are a symptom of which of the following conditions?

(a) Glaucoma

(b) Cataracts (Correct answer)

(c) Both glaucoma and cataracts

(d) None of the above

(e) I don't know

16. Red eyes are a symptom of which of the following conditions?

(a) Glaucoma (Correct answer)

(b) Cataracts

(c) Both glaucoma and cataracts

(d) None of the above

(e) I don't know

17. Excessive tearing is a symptom of which of the following conditions?

(a) Glaucoma

(b) Cataracts

(c) Both glaucoma and cataracts

(d) None of the above (Correct answer)

(e) I don't know

18. Glaucoma can lead to blindness.

(a) True (Correct answer)

(b) False

(c) I don't know

19. Cataracts can lead to blindness.

(a) True (Correct answer)

(b) False

(c) I don't know

20. Vision loss due to glaucoma can be restored.

(a) True

(b) False (Correct answer)

(c) I don't know

21. Vision loss due to cataracts can be restored.

(a) True (Correct answer)

(b) False

(c) I don't know

22. Glaucoma can be treated with medication.

(a) True (Correct answer)

(b) False

(c) I don't know

23. Cataracts can be treated with medication.

(a) True

(b) False (Correct answer)

(c) I don't know
